# eDNA Provides a Scaffold for Autoaggregation of *B. subtilis* in Bacterioplankton Suspension

**DOI:** 10.3390/microorganisms11020332

**Published:** 2023-01-28

**Authors:** Iztok Dogsa, Rok Kostanjšek, David Stopar

**Affiliations:** Biotechnical Faculty, University of Ljubljana, Jamnikarjeva 101, 1000 Ljubljana, Slovenia

**Keywords:** autoaggregates, *B. subtilis*, plankton, eDNA, biofilm

## Abstract

The self-binding of bacterial cells, or autoaggregation, is, together with surface colonization, one of the first steps in the formation of a mature biofilm. In this work, the autoaggregation of B. subtilis in dilute bacterial suspensions was studied. The dynamics of cell lysis, eDNA release, and bacterial autoaggregate assembly were determined and related to the spatial autocorrelation of bacterial cells in dilute planktonic bacterial suspensions. The non-random distribution of cells was associated with an eDNA network, which stabilized the initial bacterial cell-cell aggregates. Upon the addition of DNase I, the aggregates were dispersed. The release of eDNA during cell lysis allows for the entrapment of bacterial drifters at a radius several times the size of the dying bacteria. The size of bacterial aggregates increased from 2 to about 100 μm in diameter in dilute bacterial suspensions. The results suggest that B. subtilis cells form previously unnoticed continuum of autoaggregate structures during planktonic growth.

## 1. Introduction

In addition to adhering to inorganic and organic surfaces, bacteria also have the ability to bind to themselves. This self-binding is termed autoaggregation and is, together with surface colonization, among the first steps in the formation of a biofilm [1]. *B. subtilis* can form biofilms at the water-air interface [2]. The formation of the biofilm at the water-air interface would be facilitated if preformed buoyant autoaggregated bacterial structures float to the interface and interconnect. Currently, it is not known if *B. subtilis* can form autoaggregates in a plankton state. The driving question of this study is whether preformed autoaggregates of *B. subtilis* exist in the plankton.

Autoaggregation generally protects cells from external stresses and can be beneficial for both environmental and pathogenic bacteria, particularly under conditions such as nutrient starvation, oxidative stress, and the host immune response [3,4,5]. The molecular mechanisms underlying bacterial autoaggregation vary. Cells may autoaggregate due to electrostatic effects, hydrophobic surface properties, or through homotypic interactions between surface proteins [6,7,8,9]. Further, non-adsorbing polymers may cause bacteria to autoaggregate through depletion interactions [10]. In some cases, carbohydrates, particularly exopolysaccharides, can act as autoagglutinins. An example of an exopolysaccharide agglutinin is the polysaccharide intercellular adhesin (poly-N-acetylglucosamine; PNAG) of staphylococci [11]. A different carbohydrate-mediated autoaggregation is found in *Campylobacter jejuni*, where the autoaggregative phenotype is dependent on the glycosylation of flagella [12]. In bacteria with a charged surface, autoaggregation may start in the presence of an oppositely charged agglutinin. For example, in positively charged meningococci, an aggregate forms in the presence of polyanionic eDNA [13]. The eDNA networks may act as traps for bacteria or induce precipitation of other polymers in the suspension. For example, the extracellular DNA has been implied in the precipitation of bacterial polysaccharide levan, which formed clusters a few microns in size [14]. The eDNA in the growing planktonic culture could be a result of cell lysis [15,16], vesicle release [17], or active eDNA transport [18]. In *B. subtilis*, during pellicle formation, a substantial cell lysis occurs [19]. The eDNA is a viscoelastic molecule that aids in bacterial attachment to different surfaces and forms entangled complexes with proteins and polysaccharides [20]. The adsorption of eDNA to bacterial cell surfaces increases with increasing eDNA concentration in a solution. The adsorption increases the contact angle and the negative bacterial *ζ*-potential. Due to large size, several orders of magnitude larger than bacterial cell size, eDNA is predisposed to interconnect with neighbouring cells. It was suggested that the presence of eDNA favours bacterial aggregation via cationic bridging with Ca^2+^ at biologically relevant concentrations [20]. The eDNA was also implicated in biofilm 3D structure [21] and bacterial aggregation during the initial immune response [22,23]. High-resolution scanning electron microscopy has shown that neutrophil extracellular traps consist of stretches of DNA and globular protein domains that entrap and aggregate bacteria into structures reaching hundreds of nanometers in length and width.

In this work, we studied the formation and distribution of autoaggregates in dilute bacterial suspensions that do not show visible turbidity and sedimentation under typical aggregation assay conditions. In particular, we studied the formation of weak autoaggregated structures that could be easily disrupted during shear stress conditions used in aggregation assays (i.e., in flow cytometer or vortex assays). The long-range non-random bacterial autoaggregates were determined in real time under the microscope with spatial autocorrelation at different physiological states and cell densities. The release of eDNA during the cell lysis was observed under the fluorescence microscope. The molecular volume of the released eDNA was modelled with the string-of-beads model. The results indicate that *B. subtilis* cells form previously overlooked continuum of autoaggregate structures on eDNA scaffold during the early planktonic growth phase.

## 2. Materials and Methods

### 2.1. Bacterial Growth

The *Bacillus subtilis* NCIB 3610 wild type strain was generously provided by R. Kolter. The bacterial strain was stored at −80 °C. Prior to the experiments, it was reactivated by culturing on Lysogeny broth agar plates (LB plates; 1.0% (*w*/*v*) tryptone, 0.5% (*w*/*v*) yeast extract, 1.0% (*w*/*v*) NaCl, and 1.5% (*w*/*v*) agar) at 28 °C for 48 h. An overnight (stationary phase) culture was grown in LB growth medium at 200 r.p.m. for 16 h and washed three times by centrifugation at 10,000× *g* for 10 min (5424 Eppendorf, Hamburg, Germany). The supernatant was discarded, and the pellet was resuspended in an equal volume of saline solution (0.9% (*w*/*v*) NaCl). After the final centrifugation, the cells were resuspended in SYM medium (70 mM K_2_HPO_4_, 30 mM KH_2_PO_4_, 25 mM (NH_4_)_2_SO_4_, 0.5 mM MgSO_4_, 0.01 mM MnSO_4_, 22 mg/L ammonium iron (III) citrate, 2% (*w*/*v*) yeast extract, and 20% (*w*/*v*) sucrose). Inoculum 2% (*v*/*v*) was added to 100 mL of SYM medium in 500 mL baffled glass conical flasks and incubated at 28 °C, 200 r.p.m. For induced cell lyses experiments, we used CM medium (0.5% (*w*/*v*) glucose, 0.02% (*w*/*v*) casein hydrolysate, 0.1% (*w*/*v*) yeast extract, 2.5 mM MgCl_2,_ 78 mM K_2_HPO_4_, 44 mM KH_2_PO_4_, 15 mM (NH_4_)_2_SO_4_, 0.8 mM MgSO_4_, and 3 mM trisodium citrate.

### 2.2. Induced Cell Lysis and Visualization of Extracellular DNA

Bacterial cultures were grown to different *OD_650_* values representing exponential, early stationary, and overnight cultures at 200 r.p.m. in 100 mL baffled glass conical flasks containing 20 mL of the appropriate medium at 37 °C. Next, 2 mL of bacteria were transferred to cuvette and incubated unshaken at room temperature. *OD_650_* was measured in regular intervals with an MA 9510 photometer (Metrel, Brand, Germany). In a parallel set of experiments, 50 μL of bacterial culture were mixed with 2 μL of stock solution of TOTO-1, gently mixed, covered by 20 × 20 mm cover slide (1.5 #), and immediately transferred to the microscope. The online evolution of individual cells lysis events was monitored on DIC and fluorescence channels of Axio Observer Z1 LSM800 microscope (Zeiss, Göttingen, Germany) using × 100/1.40 NA objective operating in epi-fluorescence wide-field mode or confocal (CLSM) mode. In wide-field mode mercury, an HBO 100 W lamp, a 38 HE filter set, and an MRm Axiocam camera (Zeiss) were used. Movies were recorded using exposure times of 300 to 800 ms. In CLSM mode, a laser line 488 nm with a GaAsP PMT detector set at 700 V and with a pixel time of 0.6 μs (frame time 2.3 s) were used. The contour of the cells and the contrast in the DIC image were extracted by the aid of a custom ImageJ script. To determine eDNA concentration, bacteria were centrifuged at 10,000× *g* for 10 min to obtain the supernatant with eDNA. A concentration of ds eDNA was determined at 260 nm utilizing a Nanodrop 1000 spectrophotometer (Thermo Scientific, Waltham, MA, USA).

For DNase treatment, 1 mL of the exponential growth phase culture was concentrated 10 times and exposed to 0.2 mg/mL of DNase I from a bovine pancreas (Sigma Aldrich, St. Louis, MO, USA) and incubated at 37 °C for 30 min, followed by microscopic observation.

### 2.3. Confocal Laser Scanning Microscopy

At different time intervals during growth, 1 mL of the bacterial culture was transferred to the Eppendorf tube, stained by LIVE/DEAD (660 × diluted stock solutions), and incubated unshaken at room temperature for 0, 5, 10, 20, or 40 min before observation under an Axio Observer Z1 microscope LSM 800 (Zeiss, Göttingen, Germany) with 20 × objective (NA 0.4 and 1.6 × optovar, Zeiss) operating in confocal mode. Mosaic images corresponding to the size of 900 × 900 μm were acquired by two PMT channels set to 880 V and by two laser lines, 488 nm (Syto9) and 561 nm (propidium iodide, PI), set at 0.02% intensity. The pinhole was set to 150 μm. At least 1000 bacteria were recorded each time. For each time point, 3 separated mosaic images were recorded. The counting of bacteria was done by a custom script written in imageJ macro language. The script counts independently the bacteria stained by propidium iodide and Syto9 and detects the bacteria stained by both dyes. PI enters only cells with disintegrated membranes, whereas Syto9 can also enter the cells with intact membranes. PI-stained cells were counted as dead, while the total number of cells was determined as PI-stained + Syto9-stained cells.

### 2.4. Transmission Electron Microscopy

For negative staining transmission electron microscopy (TEM), ~20 μL of samples were transferred and left for 5 min on grids covered with Formvar support film (SPI supplies, West Chester, USA) and negatively stained with 1% (*w*/*v*) aqueous uranyl acetate for 5 s. To reduce polymeric background otherwise present in SYM media, *B. subtilis* were grown in SYM without yeast extract. The samples were fixed immediately to prevent further modifications. Excess staining solution was removed by a filter paper. Stained bacteria were air dried at room temperature and examined with a Philips CM 100 electron microscope at 80 keV.

### 2.5. Scanning Electron Microscopy

For scanning electron microscopy (SEM), the bacterial suspensions were fixed in 1% formaldehyde and 0.5% glutaraldehyde in a 0.1 M cacodylate buffer and applied to pre-cleaned glass slides. After rinsing in 0.1 M of cacodylate buffer, the attached cells were postfixed in a 1% aqueous solution of osmium tetroxide for 1 h, rinsed with water, and dehydrated in an ethanol series (50, 70, 90, 96, and 100% for 5 min each). Dehydrated samples were transferred to acetone, which was gradually replaced with hexamethyldisilazane. The samples were air dried, attached to metal holders, and sputter-coated with platinum. Prepared specimens were examined with a JSM-7500 F field emission scanning electron microscope (JEOL, Tokyo, Japan).

### 2.6. Pair-Wise Correlation Analysis of Microscopic Images

The positions of individual bacteria on microscopy images were extracted by a custom written script in ImageJ. The positional coordinates served as input parameters to a C++ program routine dedicated to calculating the spatial autocorrelation function, which calculates the normalized pair-wise correlation function defined as:

(1) $$\gamma \left(r\right)=\frac{\left\langle I\left(x\right)I\left(y\right)\right\rangle }{\left\langle I^{2}\right\rangle }$$

where *< I(x)I(y)>* is the averaged product of fluorescence intensities of two pixels at a distance *r* and <*I*^2^ > is the averaged square of intensities of all pixels, making *γ*(0) = 1. The function returns the probability of finding two bacteria at a distance *r* on the analyzed microscopy image. For a completely random position of bacteria (i.e., the density of bacteria is the same on all scales and directions), one can expect that the probability of finding two bacteria at certain distance will equal to the ratio of bacteria (non-background pixels) to the image size (all pixels):

(2) $$\gamma_{rand}\left(r\right)=\frac{N_{bacteria}}{N_{tot.\;pixels}}$$

The function is valid for *r* > 1 pixel; at *r* = 0, by definition the function returns 1. To correct for the random distribution of bacteria, we have subtracted *γ_r__and_(r)* from *γ(r)*:

(3) $$∆\gamma \left(r\right)=\gamma \left(r\right)-\gamma_{rand}\left(r\right)$$

The positive values of ∆*γ*(r) indicate a non-random distribution of bacteria. The probability to find a pair of bacteria at a certain distance is not only a function of distribution of bacteria, but also a function of a bacterial density. The more bacteria that are in the image, the more likely it is to encounter a bacterial pair at a certain distance. To account for bacterial density (i.e., exponential or early stationary culture), we have normalized the pair-wise autocorrelation function to *γ_rand_(r)* (Equation (2)):

(4) $$∆\gamma_{N}\left(r\right)=\frac{∆\gamma \left(r\right)}{\gamma_{rand}\left(r\right)}=\frac{\gamma \left(r\right)}{\gamma_{rand}\left(r\right)}-1$$

The function describes the difference in probability of finding a pair of bacteria at distance *r* in a microscopic image compared to an equal image with randomly distributed bacteria of appropriate bacterial density. If bacteria are forming aggregates, the autocorrelation function will have a positive value. The larger the value of the autocorrelation function is, the more abundant the given size of aggregated bacteria is. The size of the largest aggregated cluster of bacteria is determined as the distance *r* where the autocorrelation function crosses with the abscissa.

For spatial autocorrelation, typically 500 bacterial cells per image in the exponential phase and 2000 in the stationary phase were analyzed. We used wide-field (mosaic) images of bacteria of size 400 µm × 400 µm to obtain a representative large-scale bacterial distribution. The analyses were done on two independent samples, each consisting of at least 9 view fields. Pair-wise correlations were done for at least 10^5^ pairs. A custom C++ computer code has been used to calculate autocorrelation.

### 2.7. eDNA Modelling

For modelling eDNA with the string-of-beads model, we ran the simulation 6 times with the following parameters: number of beads = 700,000; bond angle *θ_p_* = 0; torsion angle *Φ_p_* = 0 occurring at probability of 0.9; the rest of the beads have a random torsion and bond angle with an upper boundary of 2.093 rad (for details of the simulation, please check references [24,25,26,27]).

## 3. Results

**Figure 1:**
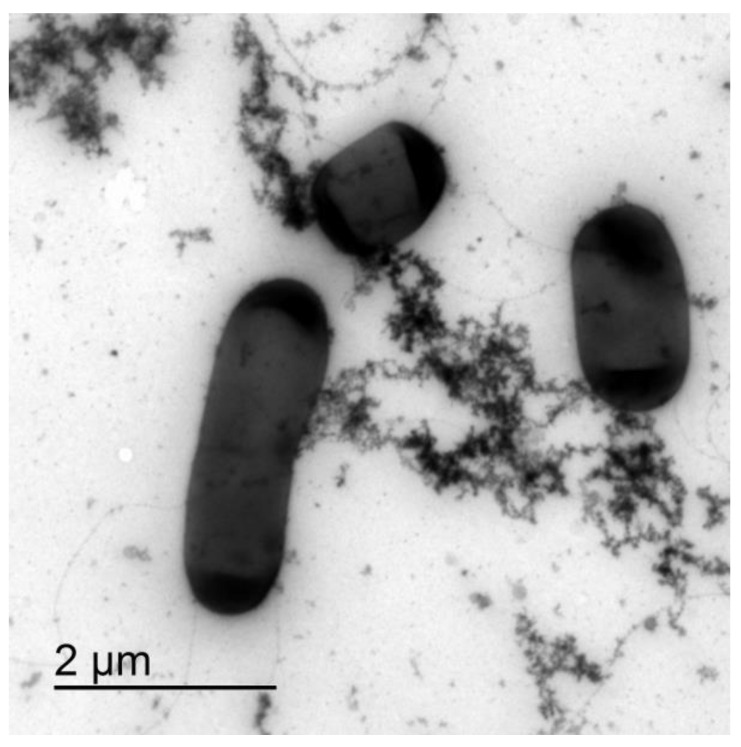
TEM of *B. subtilis* wt microcolony in the exponential growth phase after 2.5 h of incubation. Bacterial cells are interconnected via the extracellular matrix components.

At the time of inoculation, the individual bacteria were clearly visible. After two hours of incubation, flocks of extracellular material formed and some of the individual bacteria were interconnected in micro autoaggregates (Figure 1). The extracellular polymeric material interconnecting cells appeared to be rough and fractal-like. The production of extracellular material increased exponentially during the growth of *B. subtilis,* and larger interconnected bacterial autoaggregate structures formed that increased the turbidity of the suspension.

**Figure 2:**
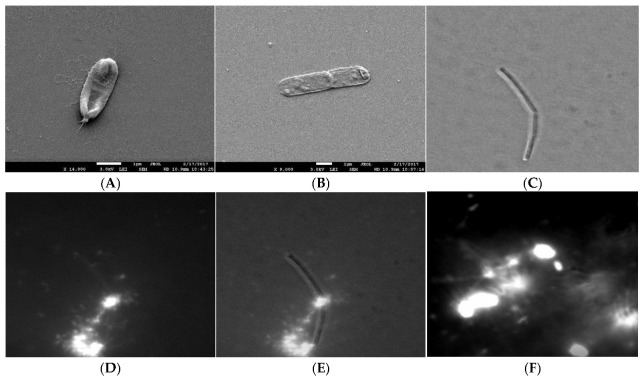
Release of cytosol material during cell lysis. (**A**,**B**) SEM micrograph of *B. subtilis* 3610 wt cells in SYM medium grown during cell lysis. The white arrow indicates rupture at the pole. Scale bar represents 1 μm. (**C**) DIC micrograph of the lysing cell, (**D**) TOTO-1 fluorescence micrograph od the same lysing cell, (**E**) an overlay of the DIC and fluorescence image. (**F**) The released fluorescence nucleic material from the dying cell (small fluorescence particles, filament structures, fuzzy cloud nucleic acid material) interconnecting the bacteria (the bright objects). To obtain the faint fluorescence structures interconnecting neighbouring bacteria, the micrographs were overexposed.

To check if the extracellular polymeric material was released during the cell lysis, we compared the electron density of the extracellular material from the mid exponentially grown bacterial culture to the material released from the induced cell lysis experiments. With TEM, it was not possible to distinguish the two materials, which suggests that at least a fraction of the extracellular material interconnecting cells in plankton could have been released during the cell lysis. Cells undergoing lysis appeared deflated on SEM and DIC micrographs (Figure 2). We observed a clear rupture of the cell structure at the pole of the bacterial cell where the cytosol material was ejected to the extracellular space. The release of the fluorescently labelled cell material during cell lysis is given in Figure 2D–F. The fluorescence material was also released from the pole of the cell. The change of morphology observed with DIC always correlated with released fluorescence material from the dying cells. The released fluorescence material was composed of discrete small particles, long filament structures radiating from the dying cell, and a fuzzy cloud of fluorescence material (Figure 2F). The numerous small fluorescence particles swarmed around the dying cell (Movie 1) and appeared to be attached to the underlying network structure. As given in Movie 2, a fuzzy cloud of extracellular fluorescent material filled the space between the dying bacteria and extended several cell diameters into the cell surrounding. The released extracellular material interconnected neighbouring cells.

**Figure 3:**
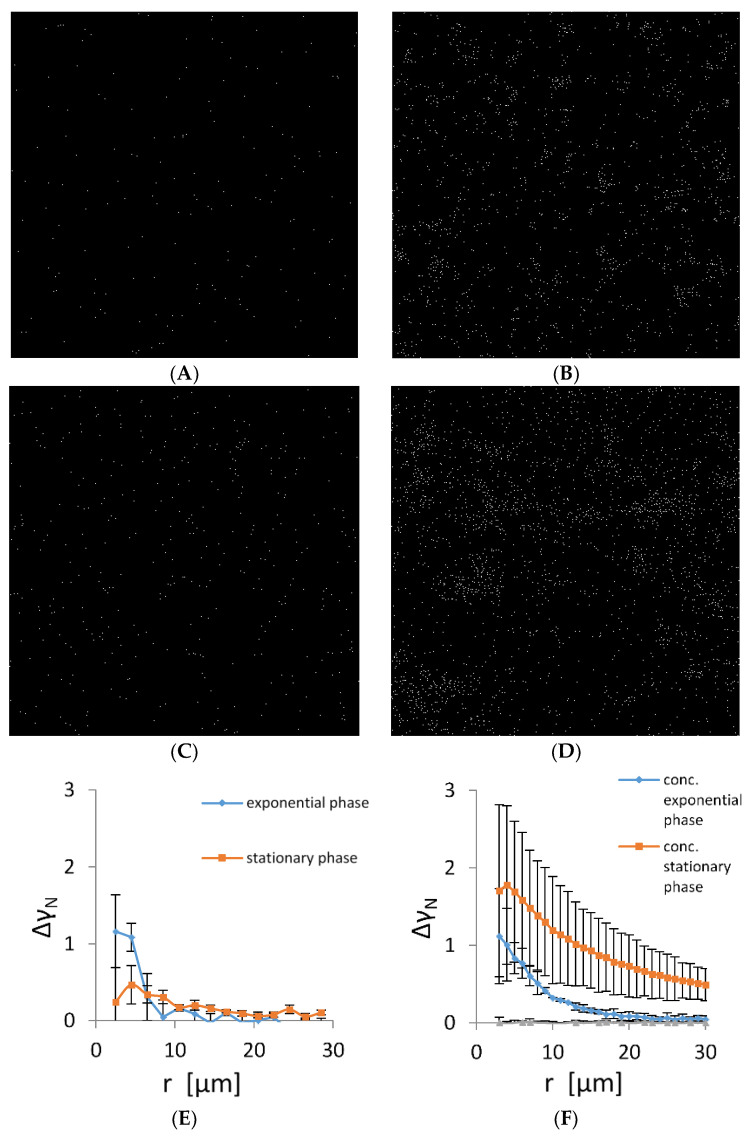
Positions of individual bacteria on microscopy images of size 400 µm × 400 µm grown in CM growth medium. (**A**) Cells in the early exponential growth phase, (**B**) early exponentially grown cells that were concentrated 5-fold, (**C**) cells in the early stationary growth phase, (**D**) the early stationary cells concentrated five folds. Scale bar corresponds to 20 µm. (**E**) Pair-wise (autocorrelation) function (Equation (3)) applied to bacterial positions in microscopy images corresponding to an area of 20 times of the areas depicted in panels (**A**–**D**). (**F**) Autocorrelation of 5-fold concentrated bacterial suspension. For comparison, a pair-wise correlation function is given for computer generated random autocorrelation function (grey line).

It is expected that cells lacking intercellular connections are randomly distributed in the plankton suspension. The TEM and fluorescence data suggest that cells autoaggregate and, therefore, are not randomly distributed. To check the randomness of the cell distribution, we determined their spatial autocorrelation in a plankton state. The representative micrographs of planktonic bacteria in the early exponential and stationary phase are given in Figure 3A,D. Early in the exponential phase, the bacterial density was low and most of the bacteria were solitary without visible contact to the neighbouring cells. However, the distribution was not random as small aggregates of up to five cells formed in the suspension. To check cell propensity for autoaggregation, we have concentrated cells 5-fold. The number of cells that autoaggregated as well as cluster long-range distribution changed significantly (Figure 3B). When cells were grown to the early stationary phase, autoaggregation increased both in non-concentrated and concentrated samples (Figure 3C,D). To quantify spatial correlations of bacterial cells, pair-wise autocorrelation was calculated (Figure 3E,F). The exponentially grown bacteria showed a significant autocorrelation at small distances, confirming the presence of small bacterial autoaggregates. The size of the autoaggregates was from 2 to 10 μm. In the early stationary phase, the correlation length extended to 20 μm, indicating that the average size of the autoaggregates increased. When the planktonic cultures were concentrated, their autocorrelation function increased significantly. For the exponentially grown cells, the size of the largest bacterial autoaggregates increased from 10 to 25 μm, whereas in the early stationary phase, the size of the largest bacterial autoaggregates increased from 20 to about 100 μm. The shape of the autocorrelation function indicates a continuous increase in size distribution of bacterial autoaggregates both in the exponential and early stationary phase.

**Figure 4:**
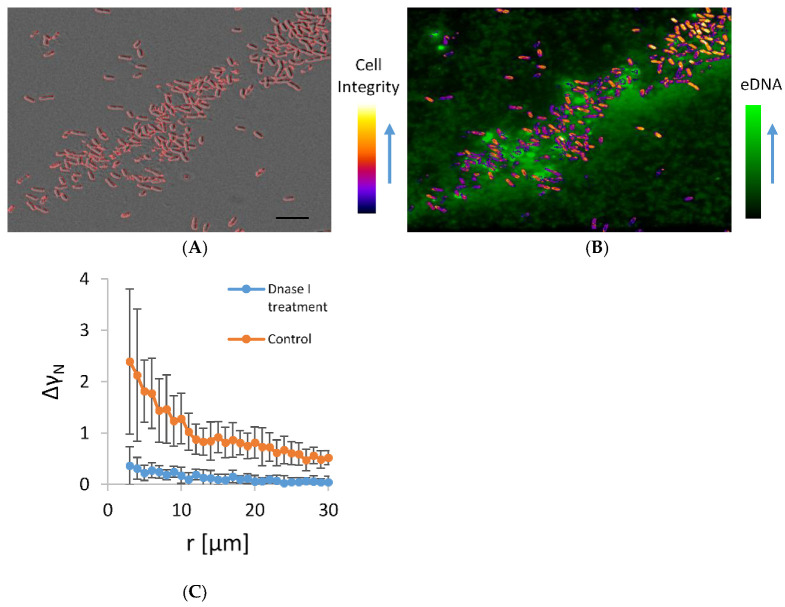
Panel (**A**) is a DIC micrograph of bacterial aggregate in the early stationary growth phase. Only high-contrast objects that include predominantly cells of high cell integrity likely to represent viable bacteria are outlined. In panel (**B**), an overlay of fluorescence image of the same bacterial aggregate stained by healthy cell impermeable DNA stain TOTO-1 and an image of colour-coded contrast intensity (Cell integrity) of DIC image is shown. Bright green fluorescence objects are the dying bacterial cells. The cells that were alive are not visible on fluorescence images and are marked by colour-coded DIC contrast intensity. Small fluorescence particles released from the dying cells swarmed around the dying cells. The scale bar corresponds to 10 µm. Panel (**C**) shows a pair-wise correlation function (Equation (3) applied on microscopy images of concentrated bacterial culture in exponential growth phase treated with DNase I for 30 min at 37 °C; control under the same conditions, but without DNase I.

The co-localization of microbial aggregates and eDNA in the early stationary phase planktonic culture are given in Figure 4. In this experiment, cells were incubated on a shaker, which exposed them to shear stress that favours only the formation of stable autoaggregates. In the aggregates, a small but significant fraction of dead cells were interdispersed among the viable cells. The viable cells in the aggregate were embedded in an eDNA network. The eDNA emanated from the dead cells and extended in the surrounding. A significant number of cells that were clearly visible on the DIC micrograph were obscured on the fluorescence image because they were embedded in a fuzzy fluorescence material of released eDNA. The viable cells were not free to move. Although some of the released fluorescence material diffused away from the point of rupture of the dying cell, the majority of the fluorescent material remained in close proximity to the dying cell, indicating the attachment to the dead cell. Some cells with intact morphology on DIC were also fluorescent, which suggests that these cells were in the process of dying and had compromised membranes. However, when DNase I was added to the culture in the exponential growth phase, such aggregates almost fully disappeared (Figure 4B), suggesting the released eDNA is the main stabilizing agent of the observed aggregates.

**Figure 5:**
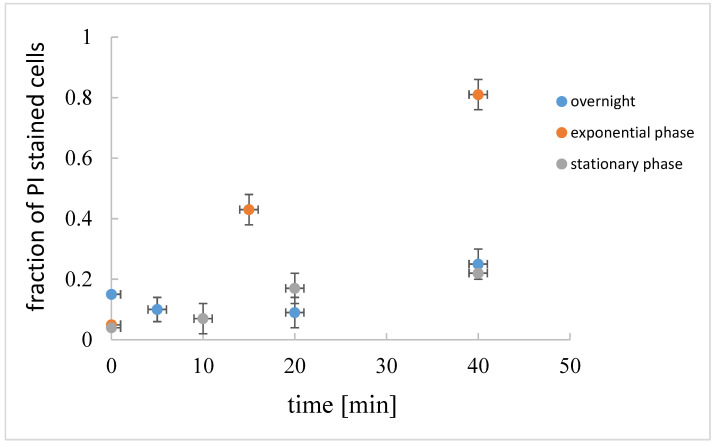
Fraction of dead cells (propidium iodide, PI, stained) in *B. subtilis* suspension at the beginning of the exponential growth (2.5 h of incubation, *OD_650_* = 0.5 a.u.), early stationary growth phase (5 h of incubation *OD_650_* = 0.6 a.u), and overnight culture (16 h of incubation, *OD_650_* = 0.8 a.u.) as determined under microscope. The fraction of dead cells in induced cell lysis at different physiological states are given at different times after cell lysis induction.

The fraction of the dead cells, i.e., the cells that are stained red by propidium iodide (PI) [28] in the exponential, stationary, and overnight culture, is given in Figure 5. In the exponential growth phase, and in the early stationary growth phase, only a small fraction of cells (up to 5%) was dead. The fraction of dead cells increased significantly in the overnight culture. Cells at different physiological states were not equally susceptible to induced cell lysis. If lysis was induced in the exponential growth phase, rapid cell lysis occurred, whereas in the early stationary growth phase or in the overnight culture, induced cell lysis was much slower.

**Figure 6:**
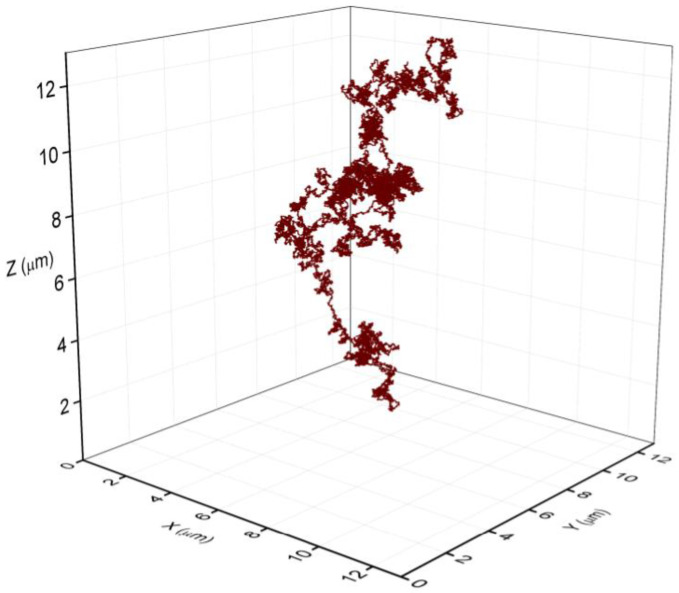
The volume space of a single eDNA released from a dying bacterium. The representative structure was obtained with a string of beads model. The scale unit on all axis is 2 μm, the average Rg of eDNA was 8 μm at persistence lenght of 50 nm [31,35]. The model eDNA was composed of 700,000 beads of 2 nm in diameter corresponding to the thickness of B-DNA and representing 4.2 Mbp of *B. subtilis* genome.

The fraction of lysing cells in the early and in the mid exponential phase was relatively small. To check if the amount of the released eDNA is capable to fill the volume of the observed bacterial aggregates, we modelled the released eDNA by the string-of-beads model [24,25,26]. The size of the DNA was 4 Mbp, and each bead corresponds to B-DNA thickness of 2 nm and the size of 6 bp [29]. The persistence length of dsDNA was assumed to be 50 nm [30,31,32,33,34,35]. Several runs of the simulation were performed, and a representative snapshot of eDNA is depicted in Figure 6. The average radius of gyration (Rg) for the eDNA was estimated to be approximately 8 μm. The effective volume occupied by eDNA scales as Rg^3^ and released eDNA molecule can fill the volume of approximately 500 μm^3^, exceeding significantly the volume of a single bacterial cell (about 1 μm^3^) facilitating local bacterial aggregation. Therefore, the released eDNA from as little as 5% of dead cells can significantly increase the connectivity between cells in the exponential grown bacterial culture and can act as agglutinin between neighbouring cells, as observed in the aggregates.

## 4. Discussion

In this work, we have demonstrated that bacterial autoaggregation, the hallmark of biofilm structure, is present in the plankton from the inoculation onwards. The size of the autoaggregates during the incubation increased from small (up to five bacteria) to large aggregates that could harbour thousands of bacterial cells. The only prerequisite for autoaggregation was the presence of extracellular material released from the naturally dying cells during population growth. This is contrary to the generally accepted view that autoaggregate formation in *B. subtilis* requires synthesis of extracellular TasA protein or at least polysaccharide EpsA-O [36,37]. Since TasA and EpsA-O are typically induced much later at the transition to the stationary growth [37,38,39], the initial autoaggregate formation must follow a different pathway. During inoculation, one does not transfer only cells but also their extracellular polymeric material. Consistently, if we have moderately concentrated diluted bacterial suspensions, the size of bacterial autoaggregates increased significantly.

From the results, it follows that an environmental perturbation that induces local order in planktonic culture correlates with the stochastic release of eDNA during cell lysis. It is reasonable to assume that the eDNA is always present in cell suspensions since a fraction of cells is always undergoing cell lysis [40,41]. The eDNA alone, or in a combination with other cellular components, was implied in the formation of aggregates in a variety of different bacteria [15,17,42,43]. The eDNA may serve as a structural component in the aggregate, as an energy and nutrition source, or a gene pool for horizontal gene transfer in naturally competent bacteria [44]. It has been suggested that small amounts of eDNA are sufficient for the development of robust biofilms [45]. Much less is known about the role of eDNA in the plankton autoaggregate formation. The eDNA released during the cell lysis in plankton remained in close contact with the dying cell and could connect neighbouring live bacteria. It has been shown that peptidoglycan, specifically *N*-acetylglucosamine, interacts with the eDNA [46]. The number of attachment sites on the cell surface is limited and can be saturated with small fragments of DNA [46]. The results of the modelling in this study suggest that eDNA can expand significantly in the extracellular medium (Figure 6). Its effective reach can be up to 500 times larger than the volume of a single bacterium. In addition, neighbouring eDNA molecules may become entangled, forming a dynamic network. The motion of the entangled eDNA is strongly hindered by the presence of the neighbouring DNA molecules, and the relaxation times may become very long and can increase from about 0.4 s in a free state to >4 s in an entangled state [47], thereby facilitating the stabilization of bacterial autoaggregates.

The presence of *B. subtilis* autoaggregates in a planktonic state at different length scales has not been observed before. From the results, it follows that the autoaggregate bacterial structures are present throughout the incubation and grow in size. The initial autoaggregates are likely fragile [38] and may quickly break under shear stress. If, on the other hand, enough extracellular material is added or dedicated extracellular material is synthesized (i.e., TasA or EpsA-O), the initial soft autoaggregates are fortified and stabilized, which allows them to grow to larger autoaggregate structures that can withstand shear stress. The larger autoaggregates may become buoyant due to gas entrapment in the viscoelastic extracellular network and float to the surface where they would form thin pellicle. The buoyancy can be increased because autoaggregates can trap CO_2_ bubbles that are the result of respiration, or bacteria can also secrete surface-active agents, such as surfactin, or synthesize a matrix that prevents mixing with the liquid medium [48,49]. Floatation can also be mediated by the attachment of the pellicle to the walls of the container near to the interface [50]. Bacteria may use more than one of these mechanisms at the same time in order to form a pellicle [51]. We argue that biofilm formation at the water-air interface is a result of autoaggregate preformation in plankton.

Plankton is an inherently non-random structure composed of free solitary drifters and a distribution of cell aggregates. The continuum distribution of autoaggregated structures in plankton is a logical consequence of continuum of organic matter in an aqueous environment. Free polymers on the scale of nm align their hydrophobic surfaces to form fibrils, which in turn entangle on a scale of 10 nm to form nanogels. Upon the annealing of nanogels, the formation of microgels takes place on the length scale from 10 to 1000 nm. Bacterial aggregation enables formation of the organic continuum on a scale from 1 to 1000 μm. Bacteria are not passive elements in the aggregate since they can actively produce and degrade extracellular material, enlarging or decreasing the size of the autoaggregate. Given bacterial exponential growth rate, the ability to autoaggregate, and the exponential production of extracellular polymer material, it is easy to see why aggregate formation in a plankton suspension may appear to pop up as an emergent structure.

A continuum of autoaggregated bacterial structures in bacterioplankton may help to explain some deep, unresolved questions in ecology, such as why social behaviour that requires critical local bacterial density may start much earlier in dilute planktonic suspensions than postulated by a standard quorum sensing model, which requires high bacterial density to allow short intercellular communication between neighbouring cells [52]. Or why oxygen-dependant production of signalling molecules is hyper-upregulated only by some cells in seemingly homogenous shaken low-density *B. subtilis* liquid cultures [53]. The local increased density due to autoaggregated bacterial structures could create low oxygen niches, triggering the expression of signalling molecule producing genes in cells in the core of the aggregate. Also, a continuum of autoaggregated bacterial structures in bacterioplankton may improve our understanding of why biogeochemical cycles are so efficient in diluted plankton communities observed in the oceans. Biogeochemical cycles rely on cross-feeding, which can be significantly enhanced in aggregated microbial communities [54]. Similarly, pre-existing bacterial microaggregates in plankton can become building blocks for the formation of pellicles and non-attached biofilms in the planktonic suspension [55].

## 5. Conclusions

The results of this study demonstrate the existence of a continuum of autoaggregated structures in *B. subtilis* bacterioplankton. The random induction of cell lysis triggers the release of eDNA, which entraps bacterial drifters at a radius several times the size of the dying bacteria. Without a further fortification of the weak eDNA viscoelastic network, the size of autoaggregates will be necessarily limited by the environmental shear stress. Specific extracellular polymeric components (i.e., TasA or EpsA-O) that *B. subtilis* produce as a response to a changing environment will strengthen the autoaggregate structure and allow their growth in size. Microbial autoaggregates that initially form stochastically as small local non-random cell distribution structures may progress to larger aggregates if cells are compatible and cooperative enough to reinforce the fragile network before it breaks. *B. subtilis* appears to have successfully solved this problem by the production of additional extracellular polymers and forms a continuous array of autoaggregated structures in plankton state not previously observed.

## Data Availability

The datasets generated during and/or analysed during the current study are available from the corresponding author.
